# Diagnostic Pitfalls in Guillain–Barré Syndrome: Case Report and Literature Review

**DOI:** 10.3390/children9121969

**Published:** 2022-12-15

**Authors:** Vasile Valeriu Lupu, Ingrith Miron, Anca Lavinia Cianga, Cristina Gavrilovici, Ioana Grigore, Alexandru Gabriel David, Leonard Iosif Pertea, Ecaterina Grigore, Diana Elena David, Ancuta Lupu

**Affiliations:** 1 Pediatrics, “Grigore T. Popa” University of Medicine and Pharmacy, 700115 Iasi, Romania; 2 “St. Mary” Children Emergency Hospital, 700309 Iasi, Romania; 3 Faculty of General Medicine, “Grigore T. Popa” University of Medicine and Pharmacy, 700115 Iasi, Romania; 4 “St. Spiridon” University Hospital, 700111 Iasi, Romania

**Keywords:** Guillain–Barré syndrome, children, meningoencephalitis, arthritis, myositis

## Abstract

Guillain–Barré syndrome (GBS) represents a group of acute immune-mediated polyradiculoneuropathies that is usually characterized by symmetrical limb weakness and areflexia. GBS can also lead to atypical clinical findings, which may lead to confusion and errors in the diagnosis. In this report, we describe a case of Guillain–Barré syndrome in a 7-year-old child who presented with neck stiffness, headache and vomiting mimicking acute meningoencephalitis, arthritis and myositis. Symptoms of ascending paralysis developed subsequently. Clearly, the atypical presentation of GBS is a significant dilemma for pediatricians and may lead to delays in diagnosis and treatment.

## 1. Introduction

Guillain–Barré syndrome (GBS) is one of the most important etiologies of flaccid paralysis; it acts on most of the spinal nerve roots and peripheral nerves [1]. It is a rare, immune-mediated disease of the peripheral nerves and nervous roots and can be fatal [2]. With a pathogenesis that is currently not fully understood, GBS may be defined as an aberrant immune response to infections that consequently damages the peripheral nerves [3]. The incidence of GBS in Europe is estimated at 1.11 per 100,000 person-years with a 20% increase for every 10-year increase in age. Furthermore, the incidence of GBS is more significant in males than in females and, clearly, it may increase during outbreaks of infectious diseases [4,5]. Regarding the age distribution of the pediatric population with GBS, many studies found a higher incidence with increasing age [6,7,8,9], but these statements were also contradicted in other studies [10,11,12].

In children, the overall incidence rates fluctuate from 0.34/100,000/year to 1.34/100,000/year [13,14]. In most cases, GBS presents with progressive limb weakness that is consecutive to an infective illness, such as upper-respiratory-tract or gastroenteric infections [15]. In the pediatric population, the rate of respiratory or gastrointestinal infections in the recent history of patients with GBS seems to be higher than in adults (Table 1) [16].

The seasonality of the disease, on the other hand, could not be established [17,18,19]. Studies focusing in this direction showed no important differences between seasons in the level of onset of GBS [20,21,22], with variations of cases clustering in winter and summer in The Netherlands [23] or in autumn in Sweden [24]. Among the triggers of GBS described in the literature, rabies and polio vaccines have been correlated with an increased risk of the disease [25,26,27,28], while for meningococcal and influenza vaccines no correlation could be made. Recently, the impact of the immunization against COVID-19 has been also studied, with arguments consequently developing for a temporal correlation between the anti-SARS-CoV-2 vaccine and the onset of GBS [29,30].

Usually, GBS occurs within 2–3 weeks of an infection. Patients may have absent reflexes or localized weakness, but also an entirely different group of clinical characteristics, delaying the diagnosis and reducing its accuracy. In children, GBS can be even more challenging because of the significant pain—muscular, radicular or neuropathic—that is usually part of the disease and which could easily mask limb weakness [31,32]. In order to establish the diagnosis of GBS, patient history, along with electrophysiological, neurological and cerebrospinal fluid (CSF) evaluations are needed. A differential diagnosis is required and diseases with similar clinical findings must be excluded as soon as possible after the onset of symptoms [33,34]. Laboratory testing usually shows a typical association of elevated CSF protein level and a normal CSF cell count, known as albumin–cytological dissociation in the cerebrospinal fluid [35,36]. Furthermore, the infiltration of macrophages, complement activation and oedema may be found among the characteristics of damaged peripheral nerves or nerve roots. Moreover, the presence of anti-ganglioside antibodies (AGAs) was also determined in the sera of GBS patients [37]. Evidence of peripheral-nervous-system dysfunction has been demonstrated through electrophysiological studies and, moreover, the subtypes of GBS, including acute motor axonal neuropathy (AMAN), acute inflammatory demyelinating polyradiculoneuropathy (AIDP), and acute motor sensory axonal neuropathy (AMSAN), can be established [38]. The absence of the H reflex is one of the most valuable parameters evaluated and it can be present from the early stage of GBS; thus, its evaluation can prevent delayed diagnoses [1].

Neuroimaging with MRI or ultrasound is recommended in order to establish the diagnosis, especially when children cannot tolerate nerve conduction studies [39]. Usually, GBS is a self-limiting disease and its recovery is spontaneous in most cases [2], but it is important to mention that GBS can progress rapidly, with many affected patients reaching their maximum disability within 14 days. Moreover, one in five patients can present signs of acute respiratory failure and may need mechanical-ventilation support. With the involvement of the autonomic nervous system, cardiac manifestations such arrythmias of fluctuant blood pressure may occur [40]. However, the emergence of immunotherapy was useful in obtaining quicker and more complete recoveries; it should be started immediately if the patient does not have the capacity to walk independently for at least 10 m, or if the patient displays rapidly progressive weakness or other severe symptoms [41]. Along with supportive care, intravenous immunoglobulin (IVIg) and plasma exchange (PE) are the two principal immunomodulatory treatment choices and they are considered to have the same efficacy in the management of GBS if started at an appropriate time [42,43]. Usually, GBS is considered a monophasic illness, but treatment-related fluctuation (TRF) must be taken into account [44]. Despite adequate therapy with IVIg or PE, approximately 20% of patients are not able to walk unaided at 6 months after disease onset and up to 10% of patients die during the disease course [45,46].

Approximately 5% of patients with GBS present relapses [47,48]. In order to establish a prognosis for GBS or the risk of a patient being unable to walk independently at 4 weeks, 3 months and 6 months after the onset of the weakness, two scores are used worldwide: the Erasmus Guillain–Barré-syndrome Outcome Score (EGOS) and the modified EGOS, which require accessible clinical elements and do not need further investigation or serological testing [49,50,51]. Poor outcomes may also be expected in patients with the need for mechanical ventilation, the axonal subtype of GBS, an advanced age at diagnosis and recent *Clostridium jejuni* infection [32]. Usually, children present milder forms of disease and better outcomes than adults with GBS [52].

## 2. Case Presentation

A 7-year-old boy presented with headache, six episodes of vomiting, one episode of fever at home, anorexia and generalized pain with predominance in the lower limbs that started 3 days before admission. He had a sore throat and stuffy nose without a high temperature 2 weeks earlier, for which he received symptomatic treatment, but these aspects were not mentioned by his mother on the day of admission.

Other than having experienced repeated upper-respiratory-tract infections for the previous 12 months, his medical history was unremarkable. He had not been vaccinated against COVID-19, nor had he received any other type of vaccine in the last year. None of his family members had experienced similar symptoms. Upon admission, both the patient and his relative were tested for COVID-19; the results were negative.

On admission, his vital signs were normal, he was afebrile and his higher mental functions were appropriate for his age. A neurological examination showed the impossibility of maximum extension at the level of the knees, pain when palpating the calves bilaterally, walking with a wide base of implantation and flexed knees, stiff neck, and plantar skin reflexes in bilateral flexion. No nystagmus, ophthalmoplegia, ataxia, or hearing loss were noticed. Deep-tendon reflexes were present and symmetrical. The results of the rest of his physical examination showed no abnormalities.

The results of his brain CT examination were normal. A lumbar puncture was also performed and the cerebrospinal fluid (CSF) analysis was not found to have been modified. The baseline blood investigations were normal and there were no markers for inflammatory syndrome. In addition, the patient had a normal renal profile, normal liver-function tests, normal thyroid function, elevated CK (three times the normal value), a normal immunoglobulin profile and a negative autoimmune screen, including double-stranded DNA, SS-A, SS-myeloperoxidase antibody, antinuclear antibodies, smooth muscle antibody and serum complement levels. The COVID-19 polymerase chain reaction was negative. Other infectious etiologies, such as *Mycoplasma pneumoniae*, mycobacterium tuberculosis, Borrelia burgdorferi, Epstein–Barr virus, cytomegalovirus and *Toxoplasma gondii* were excluded. A peripheral blood smear showed no signs of hematological malignancy or any other abnormalities.

Due to the presence of the signs of intracranial hypertension, such as neck stiffness, headache and vomiting, which mimicked acute meningoencephalitis, the patient was treated accordingly. Corticotherapy with intravenous dexamethasone and antibiotic prophylaxis with intravenous cephalosporine were initiated with the patient presenting a slow but favorable evolution.

An ensuing neurological re-evaluation found attainable orthostatism, walking with semi-flexed knees with support, bilateral symmetrical deep-tendon reflexes, absent Babinski sign and pain in the knee joint during mobilization, especially during extension. Since the CSF analysis was within the normal range, a diagnosis of arthritis was suspected. Subsequently, the patient developed functional impotence, with the inability to maintain upright posture and walking, pain in the lower limbs bilaterally and a decrease in muscle strength in the upper limbs. Gradually, the myalgia of the lower limbs aggravated and was accompanied by a decrease in strength, speed and amplitude of the segmental movements at this level. A diagnosis of infectious myositis was suggested, but the inflammatory syndrome remained absent and there were no markers of muscular lysis.

A lumbar puncture was repeated in order to assess the presence of West Nile virus, enteroviruses or mycobacterium tuberculosis, but all of the results were negative.

A psychiatric consultation was also carried out, which described the presence of emotional problems in response to the excessive conflict in the patient’s family environment. The pain-related symptoms started one day after aggressive events in the family. However, although the psychiatric examination revealed the presence of this emotional component, somatization disorder was excluded because of the characteristics of muscle pain and the attitude of the patient towards the disease.

The patient continued to complain of spontaneous muscle pain, as well as on palpation in the lower limbs. Furthermore, the patellar reflex was diminished bilaterally. Next, an immunogram was conducted and the blood complement and rheumatology panel were evaluated. They were found to be within the normal range.

Subsequently, suspecting an expansive spinal-cord process, a CT scan of the dorso-lumbar spine was also performed, which did not identify any pathological elements. An MRI could not be performed due to technical failures at the time.

Given the persistence of the patient’s symptoms, his inability to maintain the sitting position, his generalized pain, both at rest and when handled, as well as the absence of the patellar reflex bilaterally, the lumbar puncture was repeated. The cerebrospinal-fluid analysis identified albumin–cytological dissociation with a protein level of 2g/L (normal values: 0.15–0.40 g/L); combined with the patient’s clinical presentation and unremarkable CT findings, the diagnosis of Guillain–Barré syndrome was established. Unfortunately, the unavailability of autoimmune antibodies prevented us from an even stronger basis for this diagnosis.

The patient received intravenous immunoglobulin (0.4 mg/kg/day) for 5 days, along with intravenous dexamethasone, group B vitamins and physiotherapy, resulting in a favorable evolution.

One week after admission, we calculated the Modified Erasmus GBS Outcome Score (EGOS) in order to predict the probability of our patient being unable to walk independently during follow-up. The results are described in Table 2.

Two weeks after admission, at discharge, the patient’s pain subsided and his muscle strength increased significantly with active segmental movements in the lower and upper limbs, but with decreased amplitude in the lower-limb movements; the patellar reflex was still absent at the same level. Furthermore, the patient maintained the sitting position with intermittent support and refused orthostatism. Physiotherapy and supportive care were recommended at discharge.

Three days later, for the patient returned with constipation and he received symptomatic treatment. The neurological exam revealed improvements in muscle strength; the patellar reflex was still absent, but the motor deficit was decreased. The patient was able to maintain an orthostatic position and he could walk with support.

## 3. Discussion

The diagnosis of GBS is usually established according to the typical clinical features, such as paresthesia which may or may not be accompanied by symmetrical distal limb weakness weeks after an infectious illness. In clinical practice, the appearance of GBS may vary from unilateral limb weakness to facial palsy or pharyngeal weakness and diffuse sensory disturbance with ataxia, leading to difficulties in its management [53,54,55]. Patients may also present dysautonomia with heart-rate or blood-pressure instability, pupillary dysfunction, or even bladder or bowel dysfunction [31]. Moreover, Xue et al. describe in their case report an unusual onset of GBS characterized by unilateral peripheral facial paralysis, unilateral lower-limb weakness and limited outward movement of the eye [56]. In children younger than 6 years, the manifestations of GBS can be include atypical clinical features, such as failure to bear weight, irritability, localized pain with unsteady gait, or even meningism [57].

In our case, our patient’s symptoms mimicked acute meningoencephalitis, arthritis and myositis in successive phases. A spinal-cord lesion was also suspected given that, according to Ropper et al., GBS may emulate many unusual clinical conditions, including spinal lesions [58].

**Figure 1:**
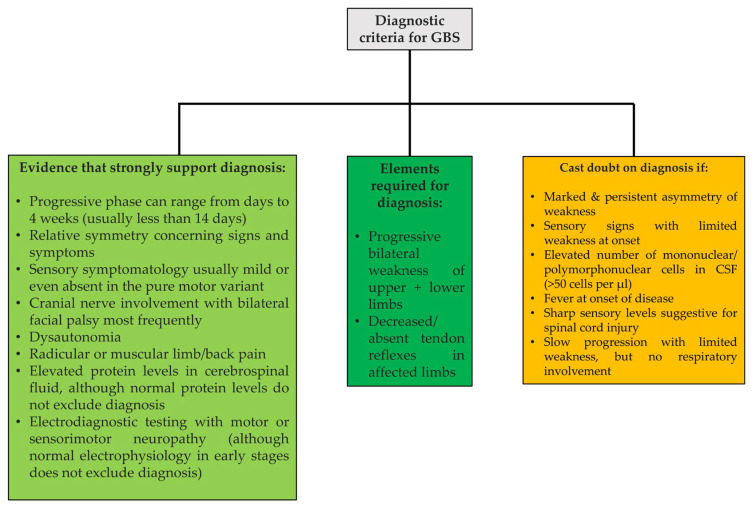
GBS diagnostic criteria [2].

The judgment and case management for our patient were made based on the diagnostic criteria for GBS developed by the National Institute of Neurological Disorders and Stroke [34,40]. We gathered and adapted these data in Figure 1, in line with Leonhard et al. [2].

A retrospective study conducted in Lebanon from 2009–2019 on 631 pediatric patients with acute flaccid paralysis showed that GBS accounted for 32.1% of the cases [59]. In order to rule out metabolic or electrolyte dysfunctions, as well as infections that can cause acute flaccid paralysis [60], we performed laboratory testing guided by the differential diagnosis with complete blood counts and studies of glycemia, electrolytes, liver and renal functions. The results of all of these measurements were negative for our patient. *Mycoplasma pneumoniae* infection, as well as *Mycobacterium tuberculosis*, *Borrelia burgdorferi*, Epstein–Barr virus, cytomegalovirus and *Toxoplasma gondii* infection [61,62,63,64,65], all known as trigger factors for GBS, could not be identified in our patient.

Moreover, given our knowledge of cases in which symmetrical polyradiculopathy was an early manifestation of acute lymphoblastic leukemia in children. which can occur even before the hematological and systemic signs [66], we performed a peripheral blood smear in order to rule out hematological malignancies.

All the other elements of the differential diagnosis taken into account in GBS are included in Table 3, in accordance with Leonhard et al. [2].

Contrary to the suggestions made by Castillo et al. [67], Gunawan et al. in their case report [68], Jaffry et al. [69] and Abolmaali et al. in their systematic review [70], we excluded the vaccine against COVID-19 as an etiological factor as the patient had not received his shot. Moreover, our patient’s history indicated repeated episodes of upper respiratory tract infections, the last of which occurred 2 weeks before admission, indicating that it was the most probable trigger of GBS in this case.

Unfortunately, our study has its limitations, such as its lack of electromyographic analysis and nerve-conduction study, as well as the technical problems that prevented us from performing an MRI. This situation prevented us from using the Brighton criteria in order to assess our patient’s disease risk [71].

Given that during the first week after disease onset, protein levels are within the normal range in 30–50% of patients and, subsequently, in 10–30% during the second week [72,73], we could not exclude the diagnosis of GBS for our patient, who presented normal CSF protein levels initially.

Although, in the literature, there are reports that describe spontaneous recovery [74], GBS is a potentially life-threatening entity and it is important to know that both medical care and immunological treatment are indispensable in managing the disease [40].

Studies claim that IVIg is efficient when started within 2 weeks of the onset of weakness and PE proves potent when started within 4 weeks [75], but we decided to use IVIg for our patient due to its greater availability and because of the reduced risk of side effects. Fortunately, our patient presented a significant improvement with the chosen treatment. However, clearly, more studies are needed in order to compare these two main options of treatment and establish whether either of them presents a greater benefit than the other. Nevertheless, IVIg or PE should be initiated immediately if a patient presents severe symptoms and has the potential for rapidly progressive weakness in order to prevent disease progression.

Furthermore, although research studies claim that the combination of IVIg and corticotherapy is not more effective than IVIg alone, our patient received both successfully. This attitude is in agreement with that of van Koningsveld et al., who believe that there might be some additional short-term effects after correction for known prognostic factors and that IVIg and corticoids might work synergistically [76].

Disease progression and outcomes are extremely inconstant in patients with GBS. Patients aged 40 years and over, patients with *Clostridium jejuni* in their medical history, along with those with significant disability at diagnosis are prone poor prognostic outcomes [77]. Although mEGOS is currently approved only for the GBS populations in Japan, Malaysia, Netherlands, [50,78] and, more recently, Europe/North America [79], we decided to calculate this score for our patient in order to assess, if possible, his prognosis. The patient had a mEGOS of 3 one week after admission and, given the favorable evolution in his condition after IVIg treatment, we suspected a good prognosis and outcome in his case.

Given that factors such as the severity of disease, autonomic dysfunction, the involvement of bulbar nerves and a longer progressive phase of the illness are associated with high mortality rates [80], GBS needs to be suspected and excluded as soon as possible after the onset of symptoms.

In a longitudinal study on 78 children from India diagnosed with GBS, Sugumar et al. found that 28.2% of patients presented neurological sequalae, while new-onset symptoms, such as frequent falls while running and fatigue, were described in 35% of children [81]. Given that the axonal variant of GBS, along with a significant GBS disability score at the point of admission are some of the predictors of neurological sequelae, we emphasize the importance of identifying these elements for the better development of a follow-up and rehabilitation plan.

Currently, research is aimed at devising a new score based on laboratory findings, such as the blood neutrophil-to-lymphocyte ratio or pre- and post-immunotherapy serum albumin and clinical aspects in order to predict the prognosis at 6 months after the onset of the disease [82].

## 4. Conclusions

Clearly, the atypical presentations of GBS is a significant dilemma for pediatricians and may lead to delays in diagnosis and treatment. The purpose of our case presentation is to highlight the fact that some symptoms mimicking meningoencephalitis, arthritis or myositis may be features of GBS at onset. Therefore, frequent reassessments and early diagnosis are of extreme importance for both patients and clinicians.

## Figures and Tables

**Table 1:** Differences between adult and pediatric populations regarding the antecedent infection rate.

	Antecedent Infection Rate	URTI	GII
Adults	40–70%	22–53%	6–26%
Children	67–85%	50–70%	7–14%

URTI = upper-respiratory-tract infection; GII = gastrointestinal infection.

**Table 2:** Results for Modified Erasmus GBS Outcome Score (EGOS) at day 7 of admission.

mEGOS Score: 3
Predicted probability of being unable to walk unaided after 4 weeks: 25%
Predicted probability of being unable to walk unaided after 3 months: 6%
Predicted probability of being unable to walk unaided after 6 months: 2%
Answers calculated to formulate result:
1. Age at onset (years)—≤40
2. Diarrhea before onset of symptoms—Absent
3. MRC sum score:—41–50

**Table 3:** Differential diagnosis of GBS.

Differential Diagnosis of GBS	
CNS	Inflammation or infection of brainstem Inflammation or infection of the spinal cord Malignancy Brainstem/spinal cord compression Brainstem stroke Vitamin deficiency
Nerve roots	Infection Compression Leptomeningeal malignancy
Peripheral nerves	Chronic inflammatory demyelinating polyradiculoneuropathy Metabolic or electrolyte disorders Vitamin deficiency Toxins Critical illness polyneuropathy Neuralgic amyotrophy Vasculitis Infection
Neuromuscular junction	Myasthenia gravis Lambert–Eaton myasthenic syndrome Neurotoxins Organophosphorate intoxication
Muscles	Metabolic or electrolyte disorders Inflammatory myositis Acute rhabdomyolysis Drug-induced toxic myopathy Mitochondrial disease
Anterior horn cells	Acute flaccid myelitis
